# McCune–Albright syndrome with multiple hyperfunctional endocrinopathies: diagnosis, treatment, and long-term follow-up: a case report

**DOI:** 10.3389/fendo.2025.1632257

**Published:** 2025-08-21

**Authors:** Mariam J. Zainab, Labibah L. Khamies, Joudi Baladi, Nawal Almutairi, Abdulhameed Alsaheel

**Affiliations:** ^1^ Alfaisal University, College of Medicine, Riyadh, Saudi Arabia; ^2^ Pediatric Endocrinology Department, Obesity, Endocrine and Metabolism Center, King Fahd Medical City, Riyadh, Saudi Arabia

**Keywords:** McCune Albright syndrome, hyperthyroidism, precocious puberty, growth hormone excess, fibrous dysplasia, lanreotide, letrozole, zoledronic acid

## Abstract

McCune–Albright syndrome (MAS) is a rare genetic disorder characterized by a triad of café-au-lait spots, fibrous dysplasia, and hyperfunctioning endocrinopathies, resulting from a mosaic mutation in the guanine nucleotide-binding protein (GNAS) gene. This case report presents the long-term follow-up of an eight-year-old girl diagnosed with MAS, who first presented at 22 months of age with skin pigmentation, hyperthyroidism, and precocious puberty, later developing additional features such as fibrous dysplasia and growth hormone excess. This complex presentation of MAS—featuring more than two hyperfunctioning endocrinopathies along with fibrous dysplasia—has rarely been described in the literature. The patient was appropriately managed with a combination of carbimazole (an imidazole), letrozole (an aromatase inhibitor), lanreotide (a somatostatin analogue), and zoledronic acid (a bisphosphonate). Notably, this is the first reported use of lanreotide in the management of MAS in a pediatric patient. This case highlights the challenges of managing MAS over an extended period, particularly when multiple endocrinopathies are present from an early age. We describe an effective approach to treatment, emphasizing how each condition was managed while considering the interactions among the various manifestations. Given the rarity of long-term follow-up reports, this case provides valuable insights into the management of MAS in pediatric patients with complex, multisystem presentations.

## 1 Introduction

McCune–Albright syndrome (MAS), a rare, sporadic disorder with an estimated prevalence ranging from 1/100,000 to 1/1,000,000, is caused by a postzygotic variant in the guanine nucleotide-binding protein (*GNAS)* gene (1). The mosaic distribution of the gene variant across different tissues leads to a wide range of symptoms, with the classic triad consisting of café-au-lait spots, polyostotic fibrous dysplasia, and hyperfunctioning endocrinopathies (1).

Fibrous dysplasia can affect any bone in the body, leading to complications such as fractures, deformities, uneven growth, scoliosis, asymmetrical facial growth, limping, and hypophosphatemic rickets (2). The café-au-lait spots in MAS exhibit distinctive characteristics: they are unilateral, do not cross the midline, and have jagged “coast of Maine” borders (1). Hyperfunctioning endocrinopathies, predominantly precocious puberty, are often the first manifestation, occurring as early as 1.8 ± 1.3 years (3). While excess growth hormone is a known feature of MAS, its co-occurrence with precocious puberty, as observed in this case, is rare. This unique combination may have significant implications for management, particularly in addressing the complex endocrine disruptions in MAS.

The diagnosis of MAS is challenging due to the mosaic nature of the *GNAS* variant, which is not always detectable by sequencing—as in our case. A clinical diagnosis is therefore critical, supported by characteristic features such as café-au-lait spots, fibrous dysplasia, and hyperfunctioning endocrinopathies. Although there is no cure for MAS, management focuses on symptom control and individualized therapy. Treatment options include bisphosphonates to reduce fracture risk, aromatase inhibitors for managing early puberty, antithyroid medications for hyperthyroidism, and surgical interventions for complications from fibrous dysplasia (2).

This case report describes a 20-month-old patient who presented with hyperactivity, irritability, palpitations, episodic vaginal bleeding since age 17 months, and progressive breast enlargement beginning at 12 months. Vital signs showed tachycardia (heart rate 140 bpm) and normal blood pressure and temperature. Upon examination, the patient had multiple unilateral café-au-lait spots, Tanner stage III breasts, Tanner stage II pubic hair, and otherwise normal systemic findings.

Initial laboratory work-up demonstrated markedly elevated estradiol (2,690 pmol/L) with suppressed LH/FSH, indicating peripheral precocious puberty; suppressed TSH (<0.01 mIU/L) with elevated free T4 (25.5 pmol/L), confirming primary hyperthyroidism; raised IGF-1 (71.15 nmol/L) and failure of GH suppression on oral glucose tolerance test (OGTT), consistent with growth hormone excess; and increased alkaline phosphatase (702 U/L), reflecting high bone turnover. Autoimmune thyroid antibodies, cortisol/ACTH, renal phosphate, and vitamin D levels were within normal limits. Imaging later revealed polyostotic fibrous dysplasia, including craniofacial involvement, supporting the diagnosis of MAS.

The co-occurrence of these features in this patient provides valuable insight into the management of MAS, particularly in the context of concurrent endocrine abnormalities. In this case, we discuss the unique clinical presentation and the management approach tailored to this patient, highlighting the interdependent nature of the various symptoms.

## 2 Patient presentation

**Figure 1:**
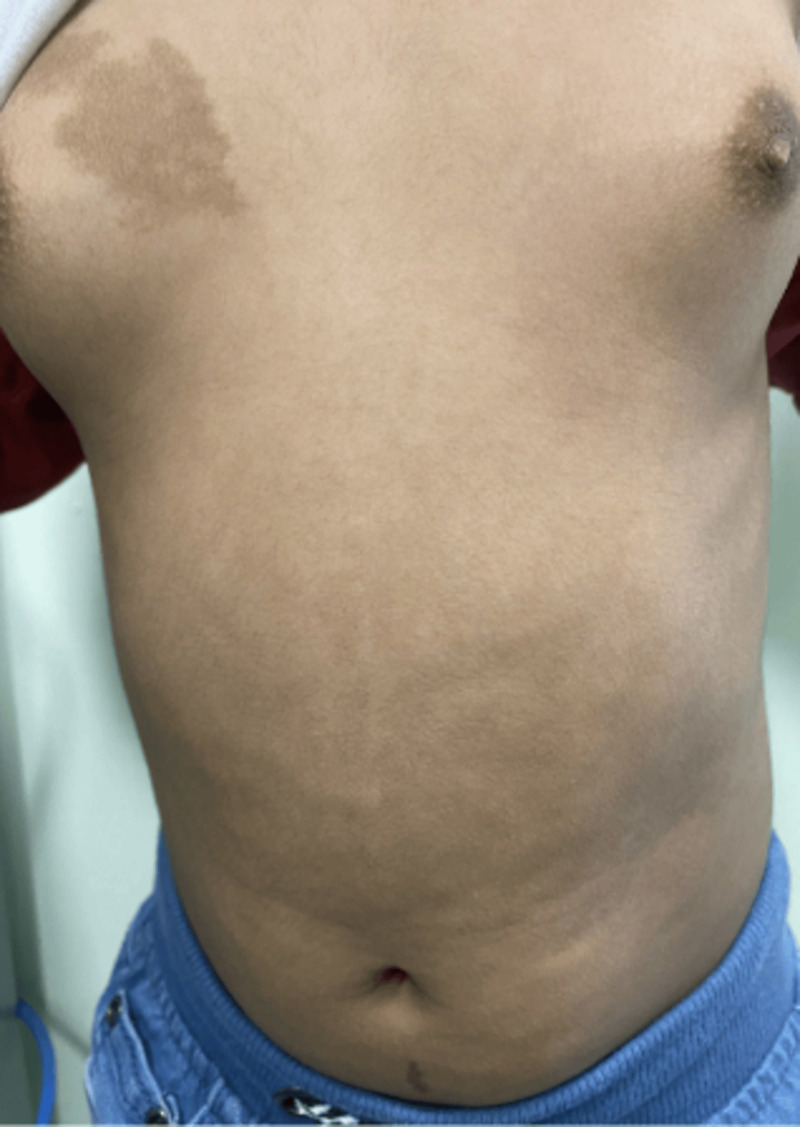
Café au lait spots: rough borders (“coast of Maine”) and did not cross the midline.

**Figure 2:**
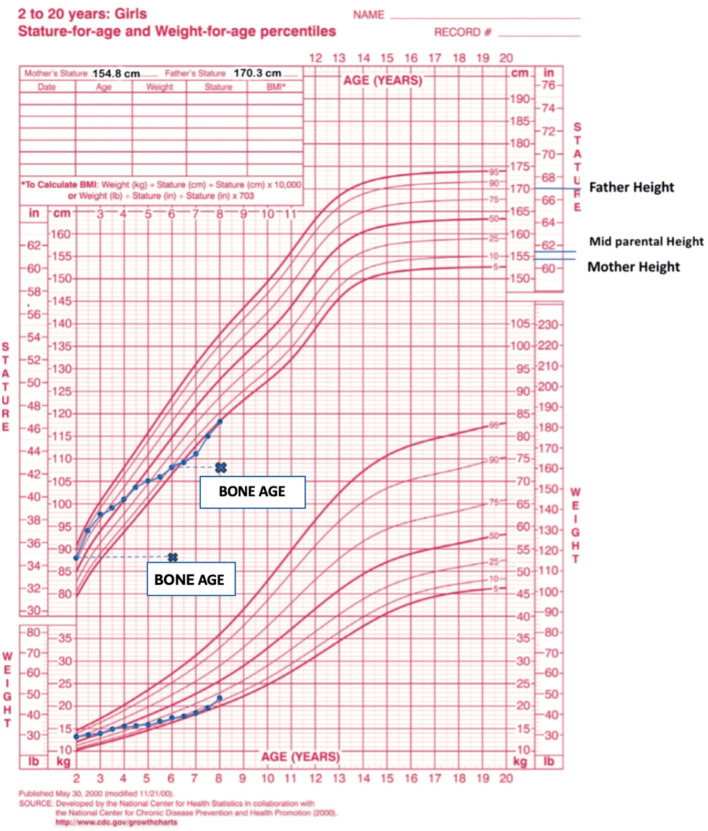
The patient’s growth chart, depicting height and weight measurements from ages 2 to 8. Data points (dots) indicate chronological age, while cross marks (x) denote bone age.

An eight-year-old girl was referred to the Pediatric Endocrinology Clinic at King Fahad Medical City (KFMC), Riyadh, Saudi Arabia, at the age of 22 months due to breast development and vaginal bleeding. The patient developed breast tissue at 12 months and experienced vaginal bleeding starting at 17 months. Breast development was gradual, painless, and without discharge. Vaginal bleeding occurred every 3 months and lasted for 3 to 7 days. In addition, the patient was hyperactive, irritable, and experienced palpitations. However, there was no associated weight loss or diarrhea. She is the only child of non-consanguineous parents. She was born full term with a birth weight of 1.8 kg (small for gestational age) and exhibited normal developmental milestones. She had a history of prolonged neonatal jaundice with elevated liver enzymes of unclear etiology, which was treated with ursodeoxycholic acid and resolved by age 2 years. A physical exam showed one café-au-lait spot on the anterior chest and multiple similar lesions on the back, which had been present since birth (**Figure 1**). Vitals at presentation showed tachycardia (heart rate 140 beats per minute), blood pressure of 119/73mmHg, and a temperature of 36.8°C. Her weight and height at presentation were 12.3 kg (50th - 75th percentile) and 87.4 cm (>75th percentile) respectively. Her mid-parental height was 156 cm (between 5th -10th centile) (**Figure 2**). The rest of the physical examination was unremarkable, except for Tanner stage II pubic hair and Tanner stage III breast development.

Given the clinical presentation, MAS was suspected. A hormonal work-up was conducted (**Table 1**), along with GNAS sequencing and radiological investigations (**Table 2**). The primary objectives were to manage the manifestations of each condition while monitoring for potential complications. Treatments administered and any subsequent changes are documented through the latest follow-up at age 8.

**Table 1:** Hormonal evaluation.

Laboratory study	At presentation	At the age of 8 years	Reference range	Interpretation
LH	<0.1 IU/L	<0.1 IU/L	<0.1 IU/L	peripheral precocious puberty (suppressed LH & FSH and very high E2)
FSH	<0.1 IU/L	0.2 IU/L	<0.1 IU/L	
E2	2690 pmol/L	<88 pmol/L	<88 pmol/L	
Random GH	3.5 ng/mL			
IGF-1	71.15 nmol/L	11.4	1.17-18.98 nmol/L	High IGF.1
OGTT for GH	(4.6/7.9/5.3/5.1) ng/ml		<0.4 ng/ml	no suppression of GH on OGTT (GH excess)
TSH	<0.01 mIU/L	2.5	0.5–5 mIU/L	Primary hyperthyroidism (suppressed TSH, high T4).
Free T4	25.5 pmol/L	10.7	9 -19	
Anti- TSH Abs, Anti-Tg Abs, Anti-TPO Abs	negative		negative	
ALP	702 U/L	350	156-369	
ALT	156 U/L	27	7 - 55	
Cortisol	231 nmol/L			Rule out Cushing syndrome.
ACTH	3.7 pmol/L		1.6-13.9	
PRL	20.5 ng/mL		5.1-26.5	
PTH	1.8 pmol/L		1.6 – 7.2	
PO4	1.43 mmol/L	1.25	> 0.36	
Tubular reabsorption of phosphate	95.7%		>78%	Rule out hypophosphatemic rickets.
25- OH vitamin D	68 nmol/L		> 75 desirable	

LH, Luteinizing hormone; FSH, Follicular stimulating hormone; E2, Estradiol; GH, Growth hormone; IGF-1, Insulin-like growth factor; OGTT, oral glucose tolerance test; TSH, thyroid stimulating hormone; Anti-Tg Ab., Anti-thyroglobulin Antibody; Anti-TPO Ab., Anti-thyroid peroxidase Antibody; ALP, Alkaline phosphatase; ALT, Alanine transaminase; ACTH, Adrenocorticotropic hormone; PRL, Prolactin; PO4, phosphorus; PTH, Parathyroid hormone.

**Table 2:** Summary of diagnostic investigations, findings, and interpretations in a 22-month-old female with suspected McCune-Albright syndrome (MAS).

Investigation	Findings	Interpretation
GNAS sequencing	Negative	Does not exclude MAS
Bone age	Six years at the chronological age of 22 months old (according to Greulich and Pyle Atlas)	Advanced bone age.
Pelvic ultrasound	Both ovaries have no cyst; the right ovary measures 1.3 cm x 1.5 cm, and the left ovary measures 2.3 x 1.9 cm. Endometrial stripe thickness measures 2 mm.	Rule out ovarian cyst causing precocious puberty.
Thyroid ultrasound	The right lobe measures 0.9 x 1.3 x 2.4 cm and the left lobe measures 0.9 x 1.4 x 3.3 cm. Both lobes demonstrate heterogeneous echo texture with increased vascularity and no focal lesion.	Rule out thyroid adenoma/hyperplasia, nodules, or enlargement.
Thyroid technetium scan	Grossly homogenous distribution of the gland with normal uptake.	Rule out homogenous high uptake (graves), and hot or cold nodules.
Brain MRI	Diffuse thickening and moderate heterogeneous enhancement of the bones of the face, the frontal bone, the skull base including the sphenoid and the temporal bones, and the occipital bone. The pituitary gland is enlarged for age, measures 5.9 mm in height with no adenoma. The posterior bright spot is normal and well-visualized.	Craniofacial fibrous dysplasia. Rule out space-occupying lesions.
Technetium bone scan	Multiple areas of increased uptake at the skull, facial bone, humerus, femur, and tibia.	Polyostotic fibrous dysplasia.

For peripheral precocious puberty, the patient was diagnosed and initiated on letrozole, an aromatase inhibitor, at a dosage of 0.8 mg orally once daily (1.5 mg/m²/day). The treatment yielded an excellent response, with regression of pubertal signs to Tanner stage I for pubic hair and stage II for breast development. Notably, estradiol levels were successfully suppressed, and her bone age was 8 years at a chronological age of 6 years.

To manage the patient’s hyperthyroidism, carbimazole was prescribed at 2.5 mg orally twice daily. The patient responded well without side effects. As medical therapy was unlikely to provide a permanent cure, total thyroidectomy was performed at age 7 years as definitive treatment. The surgery was uneventful except for transient hypoparathyroidism, which resolved within 1 week. The patient is currently maintained on L-thyroxine (37.5 mcg once daily), with normal thyroid function test results.

Following confirmation of growth hormone excess through clinical assessment and laboratory results (**Table 2**), the patient was initially treated with octreotide long-acting repeatable (LAR) at 30 mg intramuscularly once monthly. Due to the reconstitution and administration requirements of octreotide LAR, therapy was switched to lanreotide, which is provided in a prefilled syringe and administered subcutaneously. Lanreotide was started at a dose of 120 mg subcutaneously monthly and later reduced to 90 mg due to a decline in growth velocity. Monitoring insulin-like growth factor 1 (IGF-1) levels and growth rate showed normalization following treatment.

Additionally, the patient had a history of significant bone pain and recurrent fractures attributed to fibrous dysplasia. To address this, zoledronic acid was administered every 6 months. The patient demonstrated a favorable response, with substantial improvement in bone pain and a reduction in fracture episodes. Only two fractures were reported—one of which occurred following significant trauma. Frontiers style will be applied during typesetting.

## 3 Discussion

MAS has a wide spectrum of manifestations, ranging from incidental findings on imaging to debilitating diseases. The syndrome’s rarity and involvement of multiple specialties pose diagnostic challenges for clinicians. In this case, a 22-month-old girl presented with vaginal bleeding and breast enlargement, classified as Tanner stage II for pubic hair and stage III for breast development Endocrine evaluation revealed elevated estradiol levels and suppressed FSH and LH levels, consistent with peripheral precocious puberty. Physical examination also identified café-au-lait spots and symptoms of hyperthyroidism, which were confirmed biochemically as primary hyperthyroidism. These findings led to a clinical diagnosis of MAS).

MAS is diagnosed when at least two of the following are present: fibrous dysplasia of bone (FD), café-au-lait macules, and hyperfunctioning endocrinopathies. These endocrinopathies typically include gonadotropin-independent precocious puberty, hyperthyroidism, and growth hormone (GH) excess (4). Due to the postzygotic origin of the GNAS mutation and resulting mosaicism, genetic testing has limited sensitivity and specificity; false negatives may occur if unaffected tissue is sampled. Consequently, genetic testing is reserved for cases where the diagnosis cannot be made based on clinical, radiological, or histological features (4).

Precocious puberty is the most common presenting endocrinopathy in MAS and may be the only clinical manifestation. It occurs more frequently in girls, with approximately 50% of affected girls developing precocious puberty (5). Therefore, despite the rarity of MAS, it should be considered in the differential diagnosis of a young female presenting with signs and symptoms of precocious puberty (6).

Further evaluation for other hyper-functional endocrinopathies confirmed the presence of growth hormone excess and hyperthyroidism. Moreover, imaging documented craniofacial dysplasia. Growth hormone excess occurs in 20%–30% of MAS patients. While the co-occurrence of precocious puberty and growth hormone excess is rare, it presents significant clinical challenges (5). To date, 46 cases have been reported globally, with a predominance in females and frequent craniofacial complications (5). Concomitant precocious puberty and growth hormone excess synergistically accelerate linear growth velocity and advance bone age; however, this surge is transient—velocity declines sharply once bone age surpasses chronological age by >2 years (5, 7, 8). This biphasic growth pattern impairs adult height.

Diagnosis of coexisting growth hormone excess is often overlooked, not only due to the dual effects on growth but also because IGF-1—an indicator of growth hormone excess—becomes unreliable in the context of concurrent precocious puberty. IGF-1 Z-scores may reach 6.4 ± 2.1 despite normal GH secretion, mimicking the biochemical profile of pituitary gigantism (5). In addition, longitudinal data show an average 2.3-year delay in diagnosing GH excess when precocious puberty is present, as clinicians may attribute growth trends to pubertal development alone (5). Patients with elevated GH levels have a higher incidence of craniofacial FD, making GH-driven dysmorphia indistinguishable from skeletal deformities (5, 9), further delaying diagnosis. Thus, a holistic evaluation of growth velocity, bone age, and IGF-1 levels may lead to an accurate diagnosis and smooth follow-up (5). In the long run, uncontrolled precocious puberty may cause serious psychological effects in children and increase the likelihood of developing endometrial and breast cancers due to prolonged estrogen exposure. As for growth hormone excess, if left untreated, it may aggravate craniofacial fibrous dysplasia because of increased bone turnover and an increased risk of optic neuropathy and hearing loss (5). Early control of both conditions is a key pillar in management—not only to decrease growth velocity and stabilize bone maturity but also to prevent complications (5).

In this case, management of precocious puberty using letrozole—a third-generation aromatase inhibitor—yielded favorable results, including Tanner stage regression, hormone suppression, and arrest of bone age, with no complications observed. Letrozole is considered a first-line pharmacological agent for MAS-associated precocious puberty (5). Studies have concluded that it is well tolerated, with no severe adverse events, no increase in uterine volume, and no cases of ovarian torsion during treatment or follow-up (10).

For growth hormone excess, medical therapy is considered the first-line treatment, as pituitary surgery is technically challenging in MAS patients due to massive skull base thickening associated with FD (11). In our case, initial treatment was started with octreotide—a somatostatin analog. A review of 112 MAS-associated acromegaly cases (including pediatric patients) reported that somatostatin analogs achieved biochemical control in only 30% of cases, compared to 45%–50% in non-MAS pediatric acromegaly (11).

Subsequently, due to the patient’s residence in a remote area, octreotide was replaced with lanreotide, which is available in a prefilled syringe. This allowed for administration by a trained caregiver in the local area, ensuring continued treatment accessibility. Moreover, lanreotide was associated with reduced injection-related discomfort. While no pediatric-specific case studies explicitly document lanreotide use for MAS-associated GH excess, extrapolated evidence from adult MAS cases (12) and pediatric non-MAS gigantism (13) supports its potential efficacy. In our experience, the patient achieved normalization of IGF-1 levels and growth velocity without any observed complications.

FD is the most common component of MAS (14) and typically involves the craniofacial bones (11). As mentioned earlier, GH excess acts as a fuel for FD expansion (15). Similarly, treatment for precocious puberty—such as letrozole in this case—may exacerbate fibrous dysplasia. Therefore, early detection and control of GH excess in these patients is crucial (5).

Controlling GH excess with somatostatin analogs and administering zoledronic acid were successful measures taken in this case to counteract the profound effects of FD. The first clinical improvement noted with zoledronic acid was tremendous decrease in bone pain, as reported by the family. Regarding fractures, the patient experienced two during the follow-up period, one of which occurred after significant trauma.

Thyroid involvement is seen in approximately 50% of MAS patients, half of whom develop overt hyperthyroidism. It typically appears in childhood and presents with classic symptoms such as tachycardia, growth acceleration, and sleep disturbances (15, 16). Treating hyperthyroidism is particularly important in MAS patients even in the absence of clear symptoms due to its known delirious effects on bone metabolism (4). Short-term management with antithyroid medication is the first-line approach in children. However, due to its persistence into adulthood, majority of patients undergoes definitive treatment ultimately with surgical or radio-ablation therapy (15). Although the patient’s hyperthyroidism was effectively managed with carbimazole, surgical intervention was necessary, as remission is unlikely in the context of a genetic disorder. In this case, surgery was postponed due to the patient’s young age at presentation and was deferred until she reached 7 years of age.

A major dilemma for clinicians in managing MAS is the lack of unified approaches to diagnosis, treatment, and follow-up. Here, we present our experience in the long-term management of a patient with overlapping manifestations of MAS.

Due to the patient’s clinical picture, MAS was suspected. Thus, hormonal workup was performed (see **Table 1**). In addition, GNAS sequencing and radiological workup were conducted (see **Table 2**).

## Data Availability

The data that support the findings of this study are available from King Fahd Medical City portal EPIC but restrictions apply to the availability of these data, which were used under license for the current study, and so are not publicly available. Data are however available from the authors upon reasonable request and with permission of the patient and the hospital. Requests to access these datasets should be directed to dr.alsaheel@hotmail.com.
